# To Remove Impacted Cerumen

**Published:** 1901-10

**Authors:** 


### Abstracts and Selections.

### To Remove Impacted Cerumen.

In a communication to the *New York Medical Journal* Dr. E. L. Meirhof recommends highly the use of ordinary sulphuric ether to soften and assist in the removal of impacted cerumen. The ether is introduced into the external auditory canal by a suitable pipette, and after a few minutes the cerumen plug can be easily removed by gentle syringing with warm water.

